# Supplementary material for: MiR-581/SMAD7 Axis Contributes to Colorectal Cancer Metastasis: A Bioinformatic and Experimental Validation-Based Study
Source: Int J Mol Sci. 2020 Sep 5;21(18):6499. doi: 10.3390/ijms21186499 (PMC7555590; doi:10.3390/ijms21186499)
Supplement: Supplementary file 1 [file ijms-21-06499-s001.zip › Supplemental files/Supplemental Figures.pdf]

Figure S1

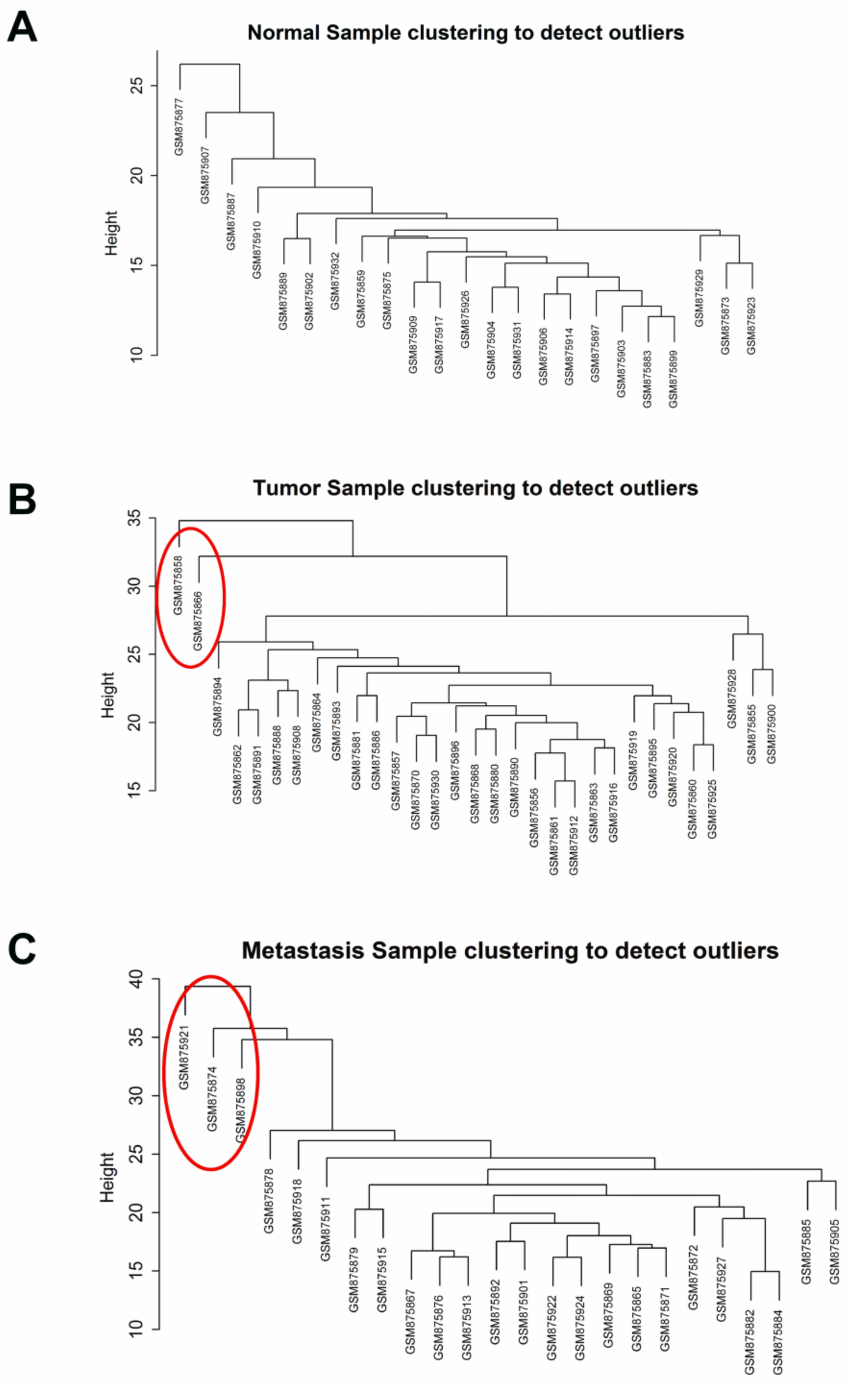

Supplementary Figure 1: Clustering dendrogram of samples for miRNA expression profiles. A, B, C are the clustering diagrams for Normal samples, Tumor samples, and

Metastasis samples of miRNA dataset, respectively. Outliers shown in B and C were removed for the following analysis.

Figure S2

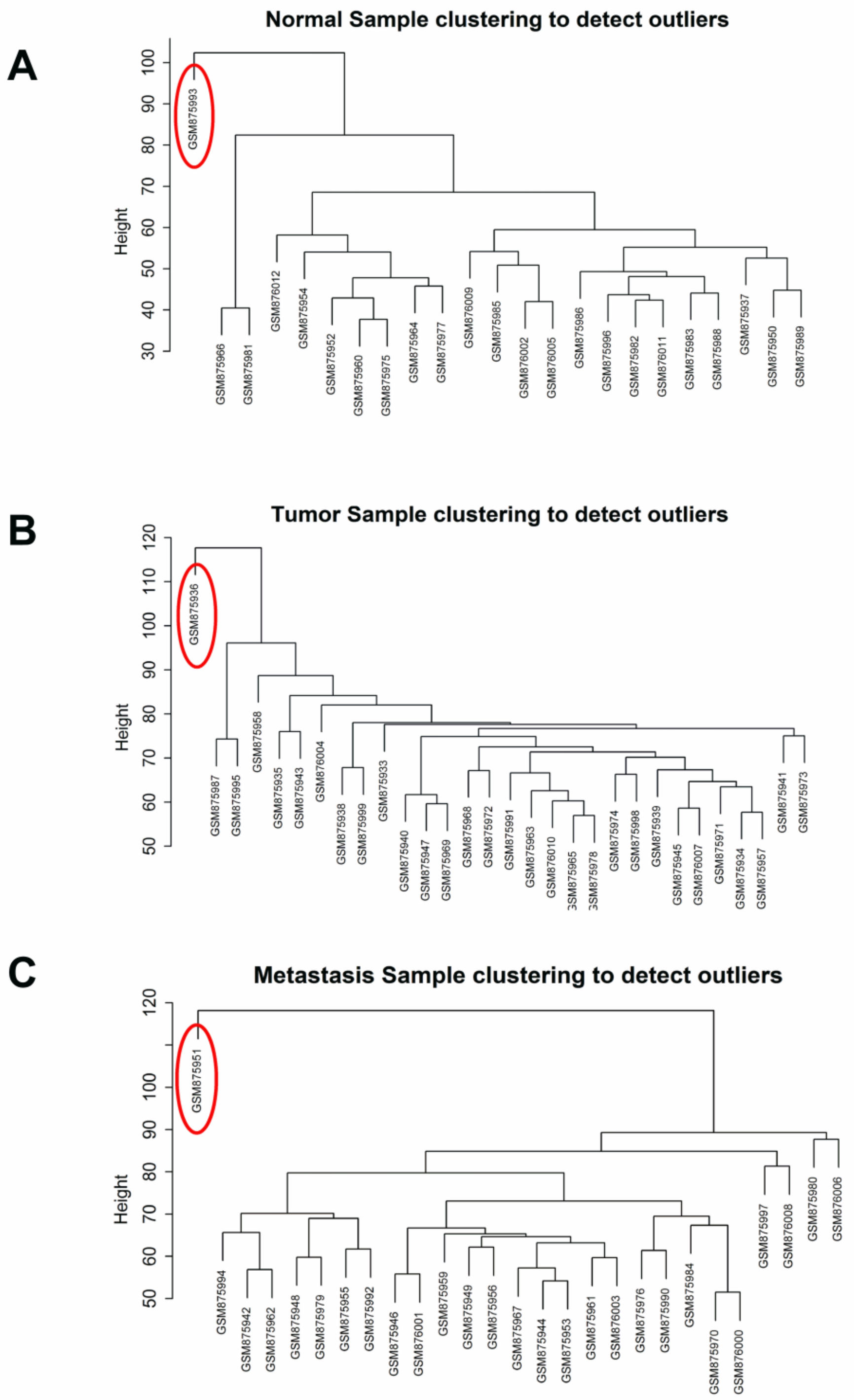

Supplementary Figure 2: Clustering dendrogram of samples for mRNA expression profiles. A, B, C are the clustering diagrams for Normal samples, Tumor samples, and

Metastasis samples of mRNA dataset, respectively. Outliers shown in A, B, and C were removed for the following analysis.

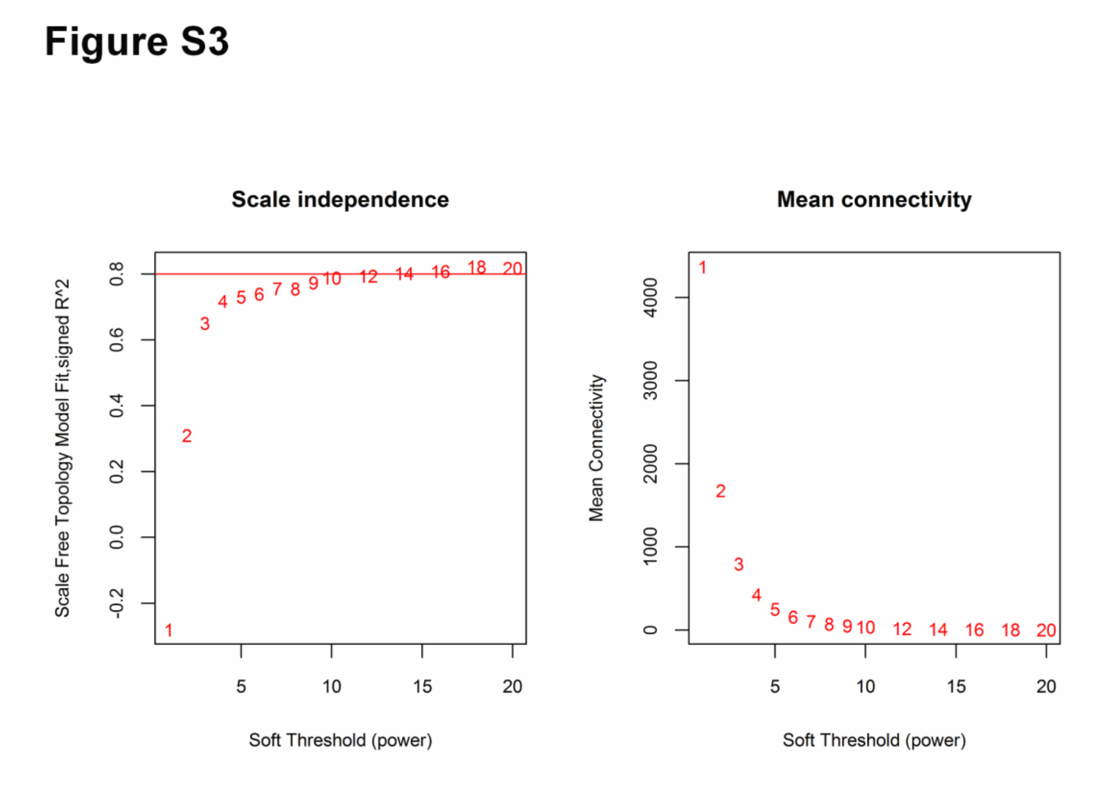

Supplementary Figure 3: Identification of soft thresholding power for network construction. The left panel shows the scale-free fit index for various soft-thresholding powers (β) whereas the right panel displays the mean connectivity for various soft-thresholding powers.

Figure S4

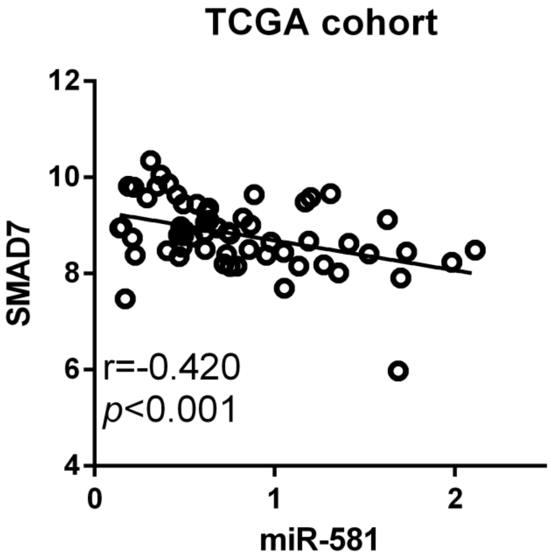

Supplementary Figure 4: miR-581 negatively correlated with SMAD7 in CRC patients with metastasis
