# Supplementary material for: MiR-581/SMAD7 Axis Contributes to Colorectal Cancer Metastasis: A Bioinformatic and Experimental Validation-Based Study
Source: Int J Mol Sci. 2020 Sep 5;21(18):6499. doi: 10.3390/ijms21186499 (PMC7555590; doi:10.3390/ijms21186499)
Supplement: Supplementary file 1 [file ijms-21-06499-s001.zip › Supplemental files/Supplemental Tables.pdf]

Supplemental Table 1: MiRNAs in profiles 6 and 7.

probeid	Profile	normal	tumor	metastases
hsa-let-7e	6	0	0.83	0.82
hsa-let-7f-2	6	0	1.17	1.21
hsa-let-7f-1	6	0	1.03	1.05
hsa-let-7g	6	0	0.86	0.55
hsa-miR-107	6	0	1.27	1.54
hsa-miR-1179	6	0	1.16	0.99
hsa-miR-1184	6	0	0.81	0.43
hsa-miR-1203	6	0	0.7	0.81
hsa-miR-1204	6	0	0.69	0.74
hsa-miR-1228	6	0	0.71	0.41
hsa-miR-1252	6	0	0.75	0.55
hsa-miR-1254	6	0	0.68	0.39
hsa-miR-1269	6	0	1.01	0.77
hsa-miR-1271	6	0	0.65	0.51
hsa-miR-1288	6	0	1.04	0.85
hsa-miR-1291	6	0	0.66	0.71
hsa-miR-1292	6	0	2.77	2.43
hsa-miR-1296	6	0	1.48	1.4
hsa-miR-1306	6	0	0.49	0.59
hsa-miR-145	6	0	0.7	0.77
hsa-miR-150	6	0	1.07	0.67
hsa-miR-151	6	0	0.98	1.21
hsa-miR-154	6	0	0.67	0.61
hsa-miR-155	6	0	1.2	1.27
hsa-miR-15a	6	0	1.37	1.51
hsa-miR-16-2	6	0	1.12	1.27
hsa-miR-16-1	6	0	1.12	1.27
hsa-miR-181d	6	0	1.6	1.54
hsa-miR-1825	6	0	2.14	2.06
hsa-miR-1827	6	0	2.11	2.24
hsa-miR-186	6	0	0.73	0.79
hsa-miR-190	6	0	1.01	0.75
hsa-miR-193b	6	0	0.98	0.78
hsa-miR-194-1	6	0	0.75	0.5
hsa-miR-194-2	6	0	0.75	0.5
hsa-miR-199a-2	6	0	0.92	0.84
hsa-miR-199a-1	6	0	0.92	0.84
hsa-miR-19b-2	6	0	0.68	0.54
hsa-miR-200a	6	0	1.81	1.45
hsa-miR-200b	6	0	1.23	1.44
hsa-miR-215	6	0	1.22	1.09
hsa-miR-216b	6	0	0.95	0.8
hsa-miR-218-2	6	0	1.27	1.19
hsa-miR-218-1	6	0	1.27	1.19
hsa-miR-220b	6	0	1.31	1.19
hsa-miR-220c	6	0	1.39	1.07
hsa-miR-224	6	0	0.94	0.81

probeid	Profile	normal	tumor	metastases
hsa-miR-26b	6	0	0.75	0.51
hsa-miR-302c	6	0	1.1	0.94
hsa-miR-324	6	0	0.88	0.83
hsa-miR-335	6	0	0.73	0.95
hsa-miR-380	6	0	0.62	0.62
hsa-miR-381	6	0	1.13	0.87
hsa-miR-382	6	0	1.25	0.73
hsa-miR-431	6	0	0.76	0.94
hsa-miR-489	6	0	1.01	0.8
hsa-miR-490	6	0	0.68	0.63
hsa-miR-492	6	0	2.24	2.94
hsa-miR-520e	6	0	0.7	0.52
hsa-miR-520f	6	0	1.09	1.15
hsa-miR-548c	6	0	0.87	0.71
hsa-miR-548f-1	6	0	1.64	2.03
hsa-miR-548f-2	6	0	1.64	2.03
hsa-miR-548f-3	6	0	1.64	2.03
hsa-miR-548f-4	6	0	1.64	2.03
hsa-miR-548f-5	6	0	1.64	2.03
hsa-miR-570	6	0	1.22	1.15
hsa-miR-578	6	0	0.93	0.51
hsa-miR-587	6	0	0.87	0.45
hsa-miR-607	6	0	0.96	0.59
hsa-miR-623	6	0	0.75	0.79
hsa-miR-624	6	0	0.55	0.64
hsa-miR-644	6	0	1.04	1.12
hsa-miR-647	6	0	0.95	1.2
hsa-miR-648	6	0	1.32	0.98
hsa-miR-651	6	0	0.84	0.73
hsa-miR-657	6	0	0.81	0.68
hsa-miR-766	6	0	0.95	0.69
hsa-miR-768	6	0	0.94	0.94
hsa-miR-886	6	0	0.87	0.69
hsa-miR-888	6	0	1.24	1.35
hsa-miR-889	6	0	0.97	0.82
hsa-miR-890	6	0	1.12	1.35
hsa-miR-891a	6	0	1.29	1.41
hsa-miR-891b	6	0	0.74	0.65
hsa-miR-892b	6	0	0.8	0.58
hsa-miR-924	6	0	1.26	1.07
hsa-miR-26a-2	6	0	1.17	0.8
hsa-miR-101-2	7	0	0.32	0.7
hsa-miR-101-1	7	0	0.32	0.7
hsa-miR-1197	7	0	0.59	0.9
hsa-miR-1206	7	0	0.78	1.13
hsa-miR-122	7	0	0.22	0.6
hsa-miR-1227	7	0	2.26	3.12

hsa-miR-26a-1	6	0	1.11	0.76
hsa-miR-1278	7	0	1.26	2.36
hsa-miR-1289-2	7	0	0.99	1.38
hsa-miR-1289-1	7	0	0.99	1.38
hsa-miR-182	7	0	0.51	0.79
hsa-miR-187	7	0	0.42	0.8
hsa-miR-22	7	0	0.51	0.72
hsa-miR-24-1	7	0	0.25	0.63
hsa-miR-24-2	7	0	0.32	0.88
hsa-miR-375	7	0	0.22	0.76
hsa-miR-377	7	0	0.69	1.03

hsa-miR-1263	7	0	1.05	1.66
hsa-miR-1280	7	0	0.42	0.59
hsa-miR-379	7	0	0.83	1.23
hsa-miR-432	7	0	0.41	0.84
hsa-miR-581	7	0	0.36	1.3
hsa-miR-598	7	0	0.61	0.84
hsa-miR-654	7	0	0.99	1.37
hsa-miR-662	7	0	0.5	0.75
hsa-miR-720	7	0	0.83	1.24
hsa-miR-767	7	0	0.72	1.51
hsa-miR-922	7	0	0.5	0.73

Supplemental Table 2: Overlapping potential target genes of alternative miRNAs.

has-miR-581			has-miR-1206				has-miR-1263		
ABCA9	KCNQ5	TNFSF10	COL4A3BP	SLF2	ARL5B	SORBS1	ZNF84	RGPD5	GALNT4
ABCG4	KITLG	TRAM2	NR5A2	TAOK1	DCUN1D4	ASAP2	ZNF763	RGPD4	FKBP7
ACKR4	KLHL15	TRIM67	NDNF	ZC3H6	CDK19	DSCC1	ZNF548	RGPD3	FGFR2
AGO1	LHFPL2	TSC22D2	NAPB	DISC1	SF3B3	ATP6AP1	ZMAT3	RCC2	FBXO46
ARHGEF6	LRRFIP1	TUBB1	GPNMB	PRRG1	PLEKHA1	ZNF367	ZEB2	RBPJ	FBXO28
ATMIN	LRRTM4	TWISTNB	FOXD4L4	GLI3	KCNJ2	NHLRC3	ZBTB26	RASSF8	FBXO11
ATP11A	LY9	UAP1L1	VSTM4	CAP2	ADCY1	HIVEP1	YAF2	RAB11A	EI24
AURKA	MAP4K4	UBP1	UBL3	BMT2	RBM25	TSC22D2	WDR41	PRRC2C	EFNA3
BAG2	MGA	UPP1	USP6	FLG2	ANOS1	NTN4	VAPA	PRPF4B	EBF2
C7orf49	MGAM	USP1	CTBP1	C9orf85	HNRNPU	RHOQ	USP8	PPP2R2C	DNAJC5G
CA10	MGME1	UST	NIPSNAP1	SCN3A	CSNK1G3	PTGES3	UQCRB	PPIP5K2	DIXDC1
CAPN2	MLLT3	WDR35	YWHAZ	FAM155A	ZMYM6	SEPT11	UBE2G2	PIGU	DAPK2
CBFA2T3	MTMR2	YWHAQ	EIF5A2	ZC3H12C	ORC4	PRKCB	TWF1	PHLDB1	CYTH3
CCDC89	MTOR	ZC2HC1A	PDCD6IP	ANKRD12	LCLAT1	NEDD4L	TRMT6	PDGFRB	CUL2
CCDC90B	MYRIP	ZFAND3	NUDT4	MFSD14A	CLVS1	ATP2A2	TRHDE	PAIP1	CSRNP1
CCKBR	NABP1	ZFP90	KCNAB3	ZNF292	UBLCP1		TOMM20L	ODF2	CSGALNACT2
CEACAM1	NALCN	ZNF138	HNMT	PPP2CA	TPK1		TMEM254	NUP50	CLU
CLEC1A	NCAN	ZNF559	SH3BGRL	KITLG	SEMA3A		TMEM245	NOTCH1	CLIP1
CNP	NR2C1	ZNF708	RIPPLY3	PI4K2B	KRAS		TMEM200A	NHLRC2	CHSY3
CTSO	NSUN7	ZNRF3	EBF3	CCDC141	MEGF11		TMEM136	NFATC1	CHST7
CUL4B	OVGP1	ZSCAN22	RHNO1	KCNB1	SERTAD4		TMEM106C	NAA30	CHD7
CYP2C18	PARPBP		SOX6	RBMS3	STXBP3		TLE3	NAA15	CCDC85A
CYTH3	PBRM1		NR1D2	P2RX2	KPNA4		TCEAL8	MTMR2	CADPS
DDHD2	PCDH7		MAP9	POMT1	MOB1B		TCEA1	MTHFD2	C6
DEFB110	PDP1		SEC23A	UBR3	ITGAV		TC2N	MFAP3L	C11orf87
DICER1	PER2		IGF2	ZBTB5	HOXA1		TASP1	MCFD2	ATP10A
DMRT3	PIK3R1		EIF3A	KCNJ15	PTPN4		STYX	MAT2A	ATAD2
DNAJC13	PIWIL1		FOSL2	TOMM70	PROS1		STX3	MAP3K2	ASAP2
DPP10	PLA2G16		EPB41L5	SAR1A	FAM199X		STX16	MAN2A1	ARIH2
DPY19L3	PLEKHM3		FUT8	LRP1B	DPP8		STK39	MAMDC2	AP3M1
EEF1A1	PNP		TIMP3	KCNB2	TBC1D19		ST8SIA4	LYPD1	ANKS1B
EPM2AIP1	QRSL1		ZNF436	SEMA6A	EPRS		SSRP1	LRTM2	ANKS1A
ERRFI1	RABL3		FOXD4L5	FAM198A	TLL2		SREBF2	LRRTM1	AKIRIN1
FAM124B	RBM15		CEMIP	MON1B	CRISPLD2		SNTB2	LGI2	AGTR2
FOXD1	RNF128		ATXN2	CSMD1	ZNF302		SNAP91	LAMC2	ACSL6
FOXO1	SHB		ZNF507	ARPC1B	DSCAML1		SMAD7	KLHL32	ABCE1
GLCCI1	SLC16A6		SMIM19	EIF1AY	ZNF805		SLC38A2	KLF4	
GNB4	SLC17A1		ESRP1	GBP6	EPHA4		SLC35F3	KIAA0040	
GPR4	SLC6A14		ZNF207	THRB	HAPLN1		SLC2A1	KCTD16	
H2AFY2	SMAD7		RREB1	FOXP2	TMEFF1		SLC25A13	KCNJ10	
HAUS2	STEAP4		ZNF718	CLIC4	ERLIN2		SLC22A7	IDUA	
HIPK3	STK36		BRINP3	KLHL2	KCNQ5		SLC22A3	HSD17B12	
HLCS	TENM1		NOVA1	IL1RAP	MTMR14		SH3KBP1	HNRNPA3	
HMGA2	TLK1		HEPN1	THSD7B	C5orf24		RPS6KA3	HMGXB4	
IMPAD1	TMEM144		YY1	CEP135	SNX18		RPL34	GYS2	
IYD	TMEM154		NRG4	GSTM4	DNAJC5		RGPD8	GNPDA1	

JAM2	TMEM47		EBPL	C8orf44	MRAS		RGPD6	GCC2	
has-miR-1278			has-miR-1289				has-miR-922		
ARID4B	KIF5B	CYP24A1	TMPRSS3	WNT1	PPA2	BSN	ABR	C10orf76	COPZ1
SMG1	HOXA5	TP53INP1	SLC6A20	TTC4	BRMS1	TPX2	ACSL1	C12orf66	CORO1C
CNOT7	RUNX1T1	PGM3	RP2	CHST5	JUNB	SLC25A18	ACVR1B	C14orf37	CPEB2
FAM126A	SEMA3E	TP53INP2	FXYD6	SLC25A41	CD2BP2		ACVR1C	C16orf87	CREBZF
WHAMM	ATP8A2	RTN1	EIF5A	EPCAM	ZNF740		ADSS	C17orf77	CRISPLD1
ERLIN2	AGAP9	RGS2	PAPD5	RBFOX2	RAB6B		AGMAT	C20orf194	CRTC1
ARG2	CNTN4	KIAA1549L	WNT2	PHC2	CHST3		AHCYL2	C2orf68	CSMD2
STARD9	NUP50	ZNF750	COPB1	TLN2	SMARCC2		AHRR	C5orf24	CSNK1G3
ARHGEF6	SAMD8	NEK7	BLCAP	B4GALNT2	HSPG2		AK3	C7orf43	CSPG5
ZCCHC14	YAF2	EEF1A1	CDKL2	CCDC71	ZRANB1		AKR7A2	CAB39	CSTF1
SERAC1	PLXDC2	CAPN6	HAND2	EFNA5	CCP110		ALDH1L2	CAB39L	CTDSP2
PANK3	TLE4	LSM14A	PON1	FHL3	CSNK1G1		ALKBH1	CABLES2	CTNNBIP1
ZNF417	PHTF1	ASXL1	MMP19	GINS1	ZFC3H1		ALOX12	CADM1	CUL2
ZBTB43	OLA1	RUFY2	HEYL	DAG1	DENND6A		AMPD3	CAMK2D	CYTIP
DNAJC6	RTF1	LRP2	ATL2	R3HDM2	AFF4		ANGEL1	CAMK2G	CYYR1
STEAP2	TACC2	SLC35A5	DDAH1	BTN3A2	HMGCS2		ANK3	CANX	DAG1
ERCC4	USP15	ZNF711	SLC35C1	AMT	ONECUT2		ANKRD13D	CARHSP1	DCAF16
RNF144B	HERPUD2	CCDC178	E2F2	CAMKMT	ATXN7		AP1G1	CASC3	DCAF5
JAK1	PSMB5	DDIT4	XRN1	TMEM207	NUAK2		AP2A1	CCDC141	DCUN1D1
RUNX2	ATP1B1	ZNF2	C22orf24	KANK4	SLC30A4		AP3M1	CCDC25	DDI2
NAA30	MTF1	AGAP4	LMO4	STAT5A	DUSP1		AP4E1	CCDC6	DERL1
EXOSC1	ALG9	LAMC1	SEMA4F	SLC2A4	SSBP4		APH1A	CCDC71L	DGKD
RCE1	DISC1	RBPJ	SLC26A6	KIFC2	SCN2A		ARCN1	CCDC88C	DICER1
POLK	C12orf65	BTBD7	DNAJB11	SOST	KRTAP2-2		AREL1	CCNG2	DKK2
AKTIP	TAOK1	SH3BGRL	KCND1	IGFBP5	CHSY1		ARF4	CCNY	DMRT2

CTAGE1	RSBN1	RPS6KB1	POU6F2	GBX2	LCE1E		ARHGEF11	CCZ1	DNAJA3
KLF3	SCN3A	KIAA0368	KCNA1	TSPAN33	GSK3B		ARID2	CCZ1B	DNM3
KCNH7	LDHAL6A	IL22	TMEM181	NFATC3	C15orf56		ARL6IP1	CDC37	DOCK5
TMEM56	TAPT1	RNPS1	KY	IRF2	PGAP1		ARL8A	CDCA4	DPF1
IQCA1	TAF5	SPTLC1	MOCS3	C5	KDM5B		ASIC1	CDCA5	DPH3
ZDHHC11	SEMA6D	PDE8A	PSME1	EIF1AX	TMEM170A		ATF1	CDH12	DPY19L4
CCNG2	CLCN5	ZNF704	SEC63	TJP2	LMX1A		ATF6	CDK17	DTX4
BMPR1A	ZNF292	EIF5B	IFIT5	PRMT3	TNFRSF10B		ATG7	CECR6	DUSP14
USP38	HOXB13	ZNF146	UACA	DUOXA2	SYS1		ATOH8	CELF3	DUSP7
RILPL1	SMIM13	THG1L	WNT4	MCL1	ATP11B		ATP10B	CELSR3	DUXA
AGAP6	ACADSB	ZKSCAN8	CGNL1	NIP7	AFF1		ATP1B3	CHMP7	EFCAB5
TMEM133	NARS2	GEN1	RSL1D1	ARHGEF2	MOGAT3		ATP2A2	CHP1	EIF2B5
RAPH1	SMAD2	CCDC14	TRIM68	FAXDC2	DAPK1		ATP2B1	CHRNA6	ELMSAN1
DOCK10	FAM193A	ABCF2	ZBTB43	MYO3A	BECN1		AVL9	CHST7	ELOVL4
QSER1	BEND6	DPY19L3	GDPD5	NAALAD2	PHYHIP		B3GNT9	CIITA	EMID1
GUCY1A3	TFPI	ENPEP	FANCC	APLN	TLE3		BACE2	CKMT2	EMILIN3
KLHL2	FXR1	RFX7	SLC30A3	BTBD9	CRB3		BAG5	CLDN18	EMP2
ZFX	SERBP1		C14orf119	METTL13	ANKRD13A		BAMBI	CLEC2B	ENDOD1
PRRC2C	AGAP5		C1orf186	RNF4	COL4A4		BANP	CLINT1	EPB41
ATRX	MYH10		STRN3	IER5L	AHCYL2		BCL6B	CLN5	ERI1
KLHL13	KLHL9		SEPT6	THEMIS	RAB23		BET1	CMTM4	ESCO1
TRIB2	APPBP2		NRP1	DESI2	XPR1		BNIP3	CNOT6L	ESR2
PPP1R15B	PALLD		ABHD14B	RPAP3	DET1		BRPF3	CNST	ESRRG
RBM12B	PHF6		SCN1A	TIMM8A	SLC4A1		BTAF1	COPS7B	ESYT3

has-miR-922								
ETV1	HAS3	KIAA0513	MKX	PAPPA	RBPMS	SMPD3	TIMP2	WNT7A

EXD2	HECTD4	KIAA1324L	MLXIP	PATL1	RC3H1	SMU1	TM9SF3	WSB2
FAF2	HEPHL1	KIAA1328	MNT	PCBP1	REEP1	SNRNP70	TMED7-TICAM2	YLPM1
FAM120A	HERC3	KIAA1456	MPC1	PCBP2	RGCC	SNX12	TMEM115	YTHDF3
FAM162A	HEY2	KIAA1549L	MPRIP	PCCA	RGS22	SNX14	TMEM30A	YWHAB
FAM189A2	HIF1AN	KIAA1644	MRPL48	PCDHA2	RHOBTB1	SNX30	TMEM74B	ZBED2
FAM53C	HIP1	KIF21B	MS4A2	PDE3B	RILPL1	SOCS1	TNPO1	ZBTB39
FAM78A	HIPK1	KIF5B	MSI2	PDLIM1	RIMKLB	SON	TNR	ZBTB40
FAM84B	HIPK3	KLC4	MTF1	PDPR	RMI2	SORT1	TP53TG3	ZBTB41
FBXL2	HLF	KLF12	MTMR12	PEX19	RNF150	SPATS2L	TPCN2	ZBTB43
FBXW7	HMG20A	KLHL28	MTMR3	PFKFB2	RNF169	SPCS2	TPPP	ZBTB44
FGFBP1	HMGB3	KPNA6	MTMR4	PFKFB3	RNF2	SPEN	TRAPPC8	ZBTB5
FGFR2	HNF4A	KRT1	MTRR	PHC1	RNFT2	SPRY3	TRIM33	ZC3H6
FHL5	HOXD13	KRT74	MTTP	PHC3	RPRD1B	SPRY4	TRIM9	ZCCHC7
FIBCD1	HPSE2	KRTAP24-1	MUM1	PI4K2B	RSAD2	SPTBN1	TRPV3	ZDHHC23
FIGN	HS2ST1	KSR2	MVB12B	PIANP	RWDD4	SPTBN2	TSC1	ZDHHC3
FKBP1A	HS3ST2	LAMC1	MYH9	PIGG	RYBP	SPTY2D1	TSPAN18	ZFHX3
FOXN2	HSPA12A	LAPTM5	MYO1C	PIGM	S1PR3	SRRM4	TSPAN6	ZFP3
FRAT2	ICK	LATS2	MYSM1	PIM1	SACM1L	SRSF6	TTC14	ZFYVE27
FUNDC1	ICOS	LCOR	NAA30	PKN2	SACS	ST5	TTC28	ZHX3
FUT10	IDH1	LGI2	NAV1	PLCG1	SAFB	STAG2	TTC33	ZNF106
FUT4	IFNAR1	LGI3	NAV2	PLD5	SARM1	STARD7	TULP4	ZNF22
FZD4	IHH	LHFPL4	NCOA3	PLEKHA8	SCD	STIM1	TYSND1	ZNF254
G3BP2	IKZF2	LIN28B	NDRG3	PLS1	SCP2	STRN	U2SURP	ZNF280D
GABPA	IL13RA1	LOR	NEK7	PLXNA4	SEC22C	STRN3	UBE2H	ZNF382
GALNT1	IL1RL2	LPP	NELFCD	POMT2	SEC31A	STX17	UBE2W	ZNF398
GALNT4	IL36RN	LRAT	NFAM1	PPA1	SEC31B	SVEP1	UBE3C	ZNF417
GAN	INO80C	LRP2	NFYB	PPARGC1B	SEMA5B	SYNC	UBFD1	ZNF474
GATAD2A	INVS	LRRC59	NMT2	PPFIBP1	SEPT6	SYT6	UBN1	ZNF585A
GCC1	IP6K1	LRRFIP2	NOMO3	PPP2R2B	SET	TAB2	UBR2	ZNF592
GGA3	IPO13	LUZP1	NPC2	PQLC3	SF1	TAF5	UBR7	ZNF608
GJA5	IRAK2	LY6G6C	NPTXR	PRKAR2A	SFTPB	TAF8	UCHL1	ZNF609
GLG1	IRF4	LYAR	NR4A2	PSD	SGSM1	TAOK1	UNC13C	ZNF706
GOLGA6L9	IRX5	LYPLA1	NR5A2	PTBP2	SH3PXD2A	TAOK3	UNC80	ZRANB2
GOLGA7	ISM2	LYSMD3	NRAS	PTER	SHOC2	TAX1BP3	UQCC1	
GOLPH3	ITGA10	MAP4	NRCAM	PTGDR	SIL1	TBCEL	UQCR10	
GOLT1B	ITIH5	MAPKAPK2	NTRK2	PWWP2A	SLC12A9	TBL1XR1	USP10	
GORASP1	ITM2B	MAPRE1	NUDT19	RAB11FIP4	SLC15A4	TCL1B	USP11	
GOT2	ITPR2	MCC	NUFIP2	RAB14	SLC22A23	TCTA	USP30	
GPD1	ITPRIP	MECP2	ODF3	RAB21	SLC23A2	TEX101	UTRN	
GPD1L	JOSD1	MED1	OPA1	RAB43	SLC24A4	TFF3	VAPB	
GPIHBP1	JOSD2	MEIS2	OSBPL2	RANBP9	SLC25A25	TGFBR1	VASH1	

GPM6B	JPH1	MERTK	OSBPL3	RAP1GAP2	SLC35B2	TGFBRAP1	VPS33A	
GPR137C	KCNE4	METTL16	OTUB2	RASA1	SLC37A4	TGIF2	VRTN	
GPR37L1	KCNIP1	METTL4	PAFAH1B1	RASSF6	SLC44A5	THBS2	VSTM4	
GRIA2	KCNJ3	MGAT4A	PAIP2B	RBM15B	SLC4A4	THY1	WASL	
GSG1	KCNK1	MICAL2	PAK2	RBM25	SLC6A13	TICAM2	WDR33	
GSK3B	KCTD1	MIEF2	PAK3	RBM34	SLC7A14	TIE1	WDR35	
HARS2	KDM2A	MKRN3	PAN3	RBMS1	SLFN5	TIGD6	WISP1	

Supplemental Table 3: Results of KEGG enrichment analysis.

<table>
<tr><th>Term</th><th>ID</th><th>Input number</th><th>Background number</th><th>P-Value</th><th>Corrected P-Value</th><th>Input</th></tr>
<tr><td>Metabolic pathways</td><td>hsa01100</td><td>1.38E+02</td><td>1.24E+03</td><td>4.33E-37</td><td>1.21E-34</td><td>229|1854|7365|5740|4723|3419|8867|1544|5980|2593|51179|8869|1373|2591|80339|3990|6999|8630|124|6718|127|1551|8877|1559|3242|6390|56922|3033|8702|51763|1558|221264|55753|5859|8608|594|120227|1329|316|132789|51251|27430|6389|8526|204|10714|10975|5091|7363|7364|4720|7366|84284|84735|8513|6675|383|10229|64850|130013|33|2572|1723|10905|79611|64902|27159|144193|23556|442117|23553|10654|570|4143|3290|10855|8942|11019|10998|1582|1581|10993|91734|51733|189|1036|79646|29958|1353|2628|2629|4717|54363|121278|10841|54802|4837|27235|55301|1109|635|4247|9104|54988|51809|9453|8564|6898|145226|5051|5053|151531|2135|112817|199857|1890|875|9517|1757|5288|23743|8399|8394|8395|54187|8790|79717|3034|1807|1040|9296|4711|5446|5444|2538|4724|1571|1576</td></tr>
<tr><td>Complement and coagulation</td><td>hsa04610</td><td>4.20E+0</td><td>7.90E+01</td><td>9.78E-34</td><td>1.37E-31</td><td>3827|629|5648|5054|7450|5624|731|2155|733|732|735|215</td></tr>
</table>

cascades		1				1|2152|2153|10747|2266|5345|2243|718|3818|721|710|2244|462|3053|5104|2158|4153|5265|5340|5327|7448|3687|3426|2147|727|722|1361|2165|2161|2160|729
Chemical carcinogenesis	hsa05204	1.90E+01	8.20E+01	9.30E-11	8.68E-09	1551|6822|1545|1559|1558|7363|7364|1544|7366|7365|405|124|64816|127|2946|1571|3290|1576|221357
Steroid hormone biosynthesis	hsa00140	1.60E+01	5.80E+01	3.20E-10	2.24E-08	1645|1551|1544|1545|7363|7364|7365|7366|8630|6718|6716|1581|1571|3290|1576|1109
Metabolism of xenobiotics by cytochrome P450	hsa00980	1.60E+01	7.30E+01	5.58E-09	3.13E-07	1645|6822|1545|1559|7363|7364|1544|7366|7365|124|127|2946|1571|3290|1576|221357
Retinol metabolism	hsa00830	1.50E+01	6.50E+01	9.15E-09	4.27E-07	1551|8608|1544|1559|1558|7363|7364|316|7366|8630|7365|124|127|145226|1576
Staphylococcus aureus infection	hsa05150	1.40E+01	5.70E+01	1.45E-08	5.78E-07	5648|4153|5340|2214|2209|629|718|3426|727|2358|721|10747|2266|2357
Drug metabolism - cytochrome P450	hsa00982	1.50E+01	6.90E+01	1.83E-08	6.41E-07	1544|7363|1559|1558|2328|7364|316|7366|7365|124|127|2946|1571|1576|221357
Bile secretion	hsa04976	1.50E+01	7.10E+01	2.55E-08	7.94E-07	6554|6579|6822|7363|10998|5244|570|6580|1244|8647|366|1581|10599|1576|10864
Tryptophan metabolism	hsa00380	1.00E+0	4.00E+01	1.47E-06	4.10E-05	1544|1545|6999|130013|8942|316|3033|8564|121278|5575

		1				3
Terpenoid backbone biosynthesis	hsa00900	8.00E+00	2.20E+01	1.64E-06	4.16E-05	23463|10654|79947|9453|57107|51449|10269|91734
Glycine, serine and threonine metabolism	hsa00260	9.00E+00	4.00E+01	1.03E-05	0.00024	635|29958|2593|2628|875|10993|64902|1757|189
Cysteine and methionine metabolism	hsa00270	9.00E+00	4.50E+01	2.34E-05	0.000503	23743|635|875|10993|4143|64902|27430|6898|1036
Drug metabolism - other enzymes	hsa00983	9.00E+00	4.60E+01	2.72E-05	0.000527	1807|151531|7363|7364|7365|7366|51733|1890|1576
PPAR signaling pathway	hsa03320	1.10E+01	7.20E+01	2.82E-05	0.000527	336|116519|10998|1582|1581|440503|345|51129|33|335|4973
Primary bile acid biosynthesis	hsa00120	6.00E+00	1.70E+01	3.91E-05	0.000651	570|10998|1582|1581|6718|1109
Biosynthesis of amino acids	hsa01230	1.10E+01	7.50E+01	3.95E-05	0.000651	5091|875|10993|4143|1373|383|229|27430|6898|5053|3419
Phagosome	hsa04145	1.60E+01	1.55E+02	4.26E-05	0.000663	10332|9341|7058|9296|2214|2209|78989|718|23673|53407|4153|4481|8685|51143|8417|4973

Huntington's disease	hsa05016	1.80E+01	1.93E+02	4.98E-05	0.000734	1213|55081|4717|836|10975|4724|6390|1329|4720|387332|3708|4723|9586|10540|4711|6389|90993|842
Parkinson's disease	hsa05012	1.50E+01	1.42E+02	5.76E-05	0.000806	4717|836|10975|2770|4724|6390|1329|4720|4723|51465|65018|11315|4711|6389|842
Non-alcoholic fatty liver disease (NAFLD)	hsa04932	1.50E+01	1.51E+02	0.000109	0.001447	4720|8503|4717|836|10975|4724|6390|1329|4217|4723|9451|1571|6389|6945|4711
Platinum drug resistance	hsa01524	1.00E+01	7.50E+01	0.000179	0.002273	4217|836|842|5980|8503|4292|4437|1244|2946|221357
PI3K-Akt signaling pathway	hsa04151	2.40E+01	3.42E+02	0.000208	0.002531	8503|2475|5156|55970|2335|3676|5586|7058|7450|2782|80310|2247|8516|3910|5894|842|2998|117145|7448|2252|2538|90993|9586|6696
Serotonergic synapse	hsa04726	1.20E+01	1.12E+02	0.000274	0.003195	836|2782|2770|1558|3708|121278|1559|3357|55970|5894|2560|9177
Carbon metabolism	hsa01200	1.20E+01	1.13E+02	0.000295	0.003304	55753|229|5091|6390|10993|51179|1373|54363|189|6389|9104|3419
Alzheimer's disease	hsa05010	1.50E+01	1.68E+02	0.000316	0.003407	4717|836|10975|6389|6390|1329|9451|4723|4720|3708|5530|4724|4711|348|842
Phosphatidylinositol signaling system	hsa04070	1.10E+0	9.80E+01	0.000339	0.003512	8867|8503|1040|5288|8394|8395|9807|3708|23262|51763|8

		1				526
Pantothenate and CoA biosynthesis	hsa00770	5.00E+00	1.80E+01	0.000445	0.004452	1807|79717|51733|79646|8876
Linoleic acid metabolism	hsa00591	6.00E+00	2.90E+01	0.000467	0.004508	1544|8399|1559|1558|1571|1576
Prostate cancer	hsa05215	1.00E+01	8.90E+01	0.000618	0.005768	8503|9586|842|2475|5156|5894|80310|6716|367|90993
Vasopressin-regulated water reabsorption	hsa04962	7.00E+00	4.40E+01	0.000649	0.005862	554|10671|4905|9586|10540|51143|90993
Pathways in cancer	hsa05200	2.50E+01	3.97E+02	0.000699	0.006118	8503|2770|2475|5156|4437|55970|836|2782|405|185|367|2247|7474|8900|9063|4313|3910|5894|842|2335|26060|4292|10928|2252|7428
Ubiquinone and other terpenoid-quinone biosynthesis	hsa00130	4.00E+00	1.10E+01	0.000747	0.006327	27235|6898|10229|3242
Insulin resistance	hsa04931	1.10E+01	1.09E+02	0.000768	0.006327	2998|8503|79660|10998|6514|2475|6195|2538|9586|90993|6945
Prion diseases	hsa05020	6.00E+00	3.50E+01	0.001116	0.008637	731|727|733|732|735|729

Oxidative phosphorylation	hsa00190	1.20E+01	1.33E+02	0.001118	0.008637	4717|9296|10975|1353|4724|6390|1329|4720|4723|27068|6389|4711
Renin secretion	hsa04924	8.00E+00	6.40E+01	0.001141	0.008637	2770|183|3708|5530|185|5137|22802|2977
ECM-receptor interaction	hsa04512	9.00E+00	8.20E+01	0.001326	0.009771	8516|7058|2335|7450|3910|7448|1605|3676|6696
Amoebiasis	hsa05146	1.00E+01	1.00E+02	0.001399	0.009867	8503|836|2335|3910|732|3458|731|733|383|735
Endocytosis	hsa04144	1.80E+01	2.60E+02	0.00141	0.009867	23327|1213|9372|58513|8394|8395|137492|64145|5156|829|11311|6457|9765|3310|8723|51699|10254|10015
TGF-beta signaling pathway	hsa04350	9.00E+00	8.40E+01	0.001546	0.010558	4086|9241|3458|9372|3626|653|9765|83729|10468
Tuberculosis	hsa05152	1.40E+01	1.79E+02	0.00162	0.010802	10332|836|842|8877|2214|2209|3458|3929|3687|5530|5894|718|26253|817
Pyrimidine metabolism	hsa00240	1.00E+01	1.05E+02	0.001954	0.012725	1807|151531|51251|1854|1723|124583|10714|221264|51733|1890
GABAergic synapse	hsa04727	9.00E+00	8.80E+01	0.002074	0.012974	2782|2770|4905|2572|2567|10991|55970|2560|6529

Phospholipase D signaling pathway	hsa04072	1.20E+01	1.44E+02	0.002085	0.012974	8503|8877|2206|8394|8395|185|2475|5156|5894|80310|554|8526
Ascorbate and aldarate metabolism	hsa00053	5.00E+00	2.70E+01	0.002147	0.013068	9104|7363|7364|7365|7366
Fat digestion and absorption	hsa04975	6.00E+00	4.10E+01	0.002299	0.013697	29881|8513|8399|335|4547|338
Neuroactive ligand-receptor interaction	hsa04080	1.80E+01	2.78E+02	0.002788	0.016264	5340|4160|554|7201|59350|84539|3357|148|2147|2151|2358|2560|2567|185|3269|83551|2357|9934
Phenylalanine metabolism	hsa00360	4.00E+00	1.70E+01	0.002852	0.016296	6898|10249|3242|5053
Regulation of actin cytoskeleton	hsa04810	1.50E+01	2.15E+02	0.003162	0.017687	8516|4633|8503|2335|8394|8395|3687|5156|2147|55970|5894|80310|2247|3676|2252
Citrate cycle (TCA cycle)	hsa00020	5.00E+00	3.00E+01	0.003222	0.017687	5091|6390|6389|55753|3419
Colorectal cancer	hsa05210	7.00E+00	6.20E+01	0.003857	0.020488	8503|836|842|26060|4292|4437|5894
Systemic lupus erythematosus	hsa05322	1.10E+01	1.36E+02	0.003878	0.020488	2214|2209|3458|718|731|727|733|732|735|721|729

Ubiquitin mediated proteolysis	hsa04120	1.10E+01	1.37E+02	0.004082	0.021167	9616|23327|9063|25898|1161|55294|26272|8452|51465|148581|7428
Calcium signaling pathway	hsa04020	1.30E+01	1.80E+02	0.004408	0.022442	8877|7201|3708|148|5156|3269|3357|5530|185|5137|490|57620|817
Valine, leucine and isoleucine degradation	hsa00280	6.00E+00	4.80E+01	0.00466	0.023026	594|5019|56922|316|3033|64902
Focal adhesion	hsa04510	1.40E+01	2.03E+02	0.004688	0.023026	8516|8503|2335|7450|3910|7448|7058|5156|4633|5906|80310|5894|3676|6696
Choline metabolism in cancer	hsa05231	9.00E+00	1.01E+02	0.004835	0.023341	8503|6580|8394|8395|2475|5156|5894|80310|8526
Glucagon signaling pathway	hsa04922	9.00E+00	1.02E+02	0.005129	0.024211	2998|6514|3708|2538|5530|9586|90993|5207|817
SNARE interactions in vesicular transport	hsa04130	5.00E+00	3.40E+01	0.005195	0.024211	55850|9341|53407|9527|8417
Long-term potentiation	hsa04720	7.00E+00	6.60E+01	0.005275	0.024211	6195|3708|5530|5894|5906|5502|817
HIF-1 signaling pathway	hsa04066	9.00E+00	1.03E+02	0.005437	0.024556	2475|3458|8503|405|7018|5054|7428|7076|817

Tyrosine metabolism	hsa00350	5.00E+00	3.50E+01	0.005797	0.025765	6898|127|124|316|3242
Amyotrophic lateral sclerosis (ALS)	hsa05014	6.00E+00	5.10E+01	0.006087	0.026181	4217|836|842|6506|5530|5608
MAPK signaling pathway	hsa04010	1.60E+01	2.55E+02	0.006128	0.026181	5608|4217|4216|836|6195|8649|55970|5156|51347|5530|5906|3310|5894|2247|9693|2252
AMPK signaling pathway	hsa04152	1.00E+01	1.25E+02	0.006171	0.026181	2998|8503|2475|148|2538|9586|90993|5207|5208|8900
cGMP-PKG signaling pathway	hsa04022	1.20E+01	1.67E+02	0.006311	0.026375	8503|2770|148|8654|3708|5530|5894|90993|185|9586|490|2977
Taurine and hypotaurine metabolism	hsa00430	3.00E+00	1.10E+01	0.006994	0.028798	1036|570|2572
Adrenergic signaling in cardiomyocytes	hsa04261	1.10E+01	1.49E+02	0.007266	0.029485	8503|2770|490|3753|148|4633|9586|90993|185|5502|817
Histidine metabolism	hsa00340	4.00E+00	2.40E+01	0.008309	0.033235	144193|10841|84735|3034
Pertussis	hsa05133	7.00E+00	7.50E+01	0.00985	0.038308	836|718|2770|710|727|722|721

Starch and sucrose metabolism	hsa00500	6.00E+0 0	5.70E+01	0.009851	0.038308	2998|7363|7364|7365|7366|2538
Pancreatic secretion	hsa04972	8.00E+0 0	9.60E+01	0.010966	0.042061	1359|8399|3708|22802|5906|1361|490|1811
Porphyrin and chlorophyll metabolism	hsa00860	5.00E+0 0	4.20E+01	0.011449	0.043322	7363|7364|1356|7366|7365
Insulin signaling pathway	hsa04910	1.00E+0 1	1.39E+02	0.011958	0.044544	2998|8503|79660|5577|122809|2475|2538|23265|5894|517 63
Inflammatory mediator regulation of TRP channels	hsa04750	8.00E+0 0	9.80E+01	0.012213	0.044544	3556|8503|3708|3357|3269|8989|5608|817
Viral myocarditis	hsa05416	6.00E+0 0	6.00E+01	0.01225	0.044544	836|842|1605|8672|6444|6443
Estrogen signaling pathway	hsa04915	8.00E+0 0	9.90E+01	0.012874	0.046216	8503|4313|2770|3708|5894|90993|9586|3310
Butanoate metabolism	hsa00650	4.00E+0 0	2.80E+01	0.013321	0.046622	5019|2572|54988|3033
Glyoxylate and dicarboxylate metabolism	hsa00630	4.00E+0 0	2.80E+01	0.013321	0.046622	189|51179|112817|54363

EGFR tyrosine kinase inhibitor resistance	hsa01521	7.00E+00	8.10E+01	0.014187	0.049041	8503|2475|5156|5894|80310|3084|2247
Platelet activation	hsa04611	9.00E+00	1.22E+02	0.014458	0.049367	8503|7450|2770|3708|5906|2244|2266|2977|2243
Peroxisome	hsa04146	7.00E+00	8.30E+01	0.015895	0.053512	10654|570|51179|92960|54363|5828|189
Phenylalanine, tyrosine and tryptophan biosynthesis	hsa00400	2.00E+00	5.00E+00	0.016054	0.053512	6898|5053
beta-Alanine metabolism	hsa00410	4.00E+00	3.10E+01	0.018118	0.059683	1807|51733|2572|84735
Amino sugar and nucleotide sugar metabolism	hsa00520	5.00E+00	4.80E+01	0.018597	0.060548	6675|8790|54187|27159|132789
Nitrogen metabolism	hsa00910	3.00E+00	1.70E+01	0.019252	0.061959	763|1373|768
Gap junction	hsa04540	7.00E+00	8.80E+01	0.020795	0.066167	2770|3708|5156|3357|5894|80310|2977
Purine metabolism	hsa00230	1.10E+01	1.76E+02	0.021256	0.066746	51251|204|5144|124583|84284|10714|22875|5137|221264|2977|8654

Arginine and proline metabolism	hsa00330	5.00E+00	5.00E+01	0.02151	0.066746	2628|383|112817|84735|2593
Fructose and mannose metabolism	hsa00051	4.00E+00	3.30E+01	0.021842	0.066746	8790|5208|5207|229
mTOR signaling pathway	hsa04150	1.00E+01	1.54E+02	0.021931	0.066746	79109|8503|9296|8649|2475|6195|5894|64121|10542|7474
Osteoclast differentiation	hsa04380	9.00E+00	1.32E+02	0.022241	0.066961	8503|2214|2209|8600|54209|5530|3458|5608|1513
Cholinergic synapse	hsa04725	8.00E+00	1.11E+02	0.022977	0.068441	8503|2782|2770|3708|55970|9586|90993|817
Morphine addiction	hsa05032	7.00E+00	9.10E+01	0.024192	0.071304	2782|2770|2560|5144|2567|55970|5137
Melanoma	hsa05218	6.00E+00	7.10E+01	0.024523	0.071315	8503|5156|5894|80310|2247|2252
Mineral absorption	hsa04978	5.00E+00	5.20E+01	0.024705	0.071315	6556|10568|490|1811|7018
Jak-STAT signaling pathway	hsa04630	1.00E+01	1.58E+02	0.025389	0.071889	8503|149233|3458|122809|316|9063|5894|2475|5771|10254

Proteoglycans in cancer	hsa05205	1.20E+01	2.05E+02	0.02547	0.071889	10855|4313|836|2335|7448|8503|3708|5894|2475|2247|7474|817
Alanine, aspartate and glutamate metabolism	hsa00250	4.00E+00	3.50E+01	0.026	0.071889	2572|1373|189|64902
African trypanosomiasis	hsa05143	4.00E+00	3.50E+01	0.026	0.071889	335|3250|3458|3910
Glutamatergic synapse	hsa04724	8.00E+00	1.14E+02	0.026188	0.071889	2782|2770|6507|55970|6506|10991|3708|5530
Fc gamma R-mediated phagocytosis	hsa04666	7.00E+00	9.30E+01	0.026658	0.072468	8503|8877|2214|8394|8395|5894|2209
Pentose and glucuronate interconversions	hsa00040	4.00E+00	3.60E+01	0.028244	0.076042	7363|7364|7365|7366
Arrhythmogenic right ventricular cardiomyopathy (ARVC)	hsa05412	6.00E+00	7.40E+01	0.028925	0.077134	8516|1605|6444|6443|3676|1000
Rap1 signaling pathway	hsa04015	1.20E+01	2.11E+02	0.030548	0.080132	5608|8503|2252|2357|2770|5156|5906|80310|5894|2247|9693|9855
Chemokine signaling pathway	hsa04062	1.10E+01	1.87E+02	0.030622	0.080132	6354|2782|2770|8503|2268|55970|5906|9547|5894|6360|56477

Toxoplasmosis	hsa05145	8.00E+00	1.19E+02	0.032212	0.083512	8503|836|842|3910|2770|3458|3310|5608
Protein processing in endoplasmic reticulum	hsa04141	1.00E+01	1.66E+02	0.033448	0.085922	4217|4287|27248|9451|10905|51465|81567|3310|80267|10427
Olfactory transduction	hsa04740	2.00E+01	4.20E+02	0.033889	0.086263	2782|343406|390442|23538|390261|390054|122740|341152|26735|4992|138803|390892|5137|26659|219453|390148|119678|254783|219431|817
Hepatitis B	hsa05161	9.00E+00	1.46E+02	0.037618	0.094078	8503|836|842|5894|90993|10542|9586|1959|8900
AGE-RAGE signaling pathway in diabetic complications	hsa04933	7.00E+00	1.01E+02	0.038214	0.094078	8503|4313|836|2335|2152|185|5054
Biosynthesis of unsaturated fatty acids	hsa01040	3.00E+00	2.30E+01	0.038639	0.094078	570|54898|79071
Renin-angiotensin system	hsa04614	3.00E+00	2.30E+01	0.038639	0.094078	183|1359|185
Mismatch repair	hsa03430	3.00E+00	2.30E+01	0.038639	0.094078	10714|4437|4292
VEGF signaling pathway	hsa04370	5.00E+0	6.10E+01	0.042755	0.103201	8503|5530|5894|842|8877

		0				
Hypertrophic cardiomyopathy (HCM)	hsa05410	6.00E+0<br>0	8.30E+01	0.045128	0.107121	8516|1605|4633|6444|6443|3676
Arachidonic acid metabolism	hsa00590	5.00E+0<br>0	6.20E+01	0.045144	0.107121	1559|1558|1571|8399|5740
Fatty acid elongation	hsa00062	3.00E+0<br>0	2.50E+01	0.046668	0.109808	54898|79071|3033
Dopaminergic synapse	hsa04728	8.00E+0<br>0	1.30E+02	0.048627	0.113464	2782|2770|55970|3708|5530|9586|90993|817
